# Feasibility of a Proactive Text Messaging Intervention for Smokers in Community Health Centers: Pilot Study

**DOI:** 10.2196/formative.9608

**Published:** 2018-05-31

**Authors:** Gina Kruse, Jennifer HK Kelley, Karen Chase, Nancy A Rigotti

**Affiliations:** ^1^ Division of General Internal Medicine Massachusetts General Hospital Boston, MA United States; ^2^ Tobacco Research and Treatment Center Massachusetts General Hospital Boston, MA United States; ^3^ Harvard Medical School Boston, MA United States; ^4^ Partners Center for Connected Health Partners HealthCare Somerville, MA United States

**Keywords:** smoking cessation, primary health care, text messaging

## Abstract

**Background:**

Few smokers receive evidence-based cessation services during primary care visits.

**Objective:**

We aimed to assess the feasibility of a proactive text messaging program for primary care patients who smoke.

**Methods:**

We used electronic health records to identify smokers who had a mobile phone number listed from two community health centers in Massachusetts. Between March 2014 and June 2015, patients were screened by their primary care physician and then sent a proactive text message inviting them to enroll by texting back. Patients who opted in were asked about their readiness to quit. The text message program included messages from the QuitNowTXT library and novel content for smokers who were not ready to quit.

**Results:**

Among 949 eligible smokers, 88 (9.3%) enrolled after receiving a single proactive text message. Compared with those who did not enroll, enrollees were more often female (54/88, 61% vs 413/861, 48.0%, *P*=.02), but otherwise did not differ in age, race, insurance status, or comorbidities. In all, 28% (19/67) of enrollees reported they were not ready to quit in the next 30 days, 61% (41/67) were ready to quit, and 11% (7/67) already quit. The median time in the program was 9 days (interquartile range 2-32 days). Of current smokers, 25% (15/60) sent one or more keyword requests to the server. These did not differ by readiness to quit.

**Conclusions:**

A proactively delivered text messaging program targeting primary care patients who smoke was feasible and engaged both smokers ready to quit and those not ready to quit. This method shows promise as part of a population health model for addressing tobacco use outside of the primary care office.

## Introduction

Among US smokers, less than one-third use any assistance—pharmacologic or behavioral—when they try to quit smoking [1]. Text messaging shows promise as a way to assist smokers to quit by delivering behavioral advice. Prior studies indicate that text messaging interventions for smokers increase tobacco abstinence rates by 36% to 70% [2-13]. However, most prior text messaging studies recruited motivated smokers through public advertisements, the Internet, or school-based recruitment. The few text messaging studies that recruited smokers from health care settings targeted motivated smokers [14], those already in tobacco treatment programs [15], pregnant smokers [16], or patients with coronary disease [17]. The feasibility of delivering tobacco cessation assistance by text message for the broader population of smokers in primary care is unknown.

Primary care practices are well positioned to promote smoking cessation because 70% of smokers visit a physician each year [18]. However, although physicians often recommend quitting during visits, competing priorities and time constraints prevent them from offering further assistance [19]. Thus, new proactive models of care delivery are being developed for smokers [20-26]. These programs reach out to patients who are listed as smokers in electronic health records (EHRs) between visits to offer them help [27]. Prior models using mailings and telephone calls to engage smokers produced increases in treatment use and tobacco abstinence [20-26]. Text messaging interventions may be a less costly way to increase the reach and engagement of smokers in these proactive models [28].

Proactive models allow health systems to reach out to all smokers, not just those seeking treatment. In the United States, 80% of smokers are not ready to quit in the next 30 days [29]. However, smokers who are not ready to quit report substantial interest in mobile health interventions [30]. A low-intensity intervention such as text messaging may be a better fit with the treatment preferences of smokers who are not ready to quit compared to more intensive or intrusive treatments. Furthermore, even moderately efficacious interventions that target the large proportion of smokers who are not ready to quit may have a large public health impact [31]. The objective of this study was to assess the feasibility of delivering a proactive text messaging intervention for smokers in primary care in terms of proportion of patients reached, their interaction, and duration of time spent with the program.

## Methods

### Participants

We recruited smokers receiving primary care at Massachusetts General Hospital-affiliated community health centers in Charlestown and Revere, Massachusetts. We identified patients receiving primary care using a validated algorithm used by the Massachusetts General Hospital Practice-Based Research Network [32]. Other eligibility criteria based on EHR data included a primary language of English, age 18 years and older, current smoker, and with a mobile telephone number.

### Intervention

We developed the GetReady2Quit (R2Q) text messaging program with content for smokers ready to quit in the next 30 days and content for smokers not ready to quit (Table 1). For smokers who were ready to quit in the next 30 days, we used the downloadable QuitNowTXT library [33]. QuitNowTXT includes 118 messages delivered over 6 weeks tailored to a user-entered quit date. These messages include behavioral advice and motivational and educational messages about the harms of tobacco and the benefits of quitting. The program has limited two-way communication including keywords to request help by texting “CRAVE,” “MOOD,” or “SLIP.” There were also weekly smoking status assessment messages that invited a user response. Unlike other text messaging programs [34], if a user did not respond to an assessment, no further messages were sent. Maximum message volume was 25 messages per week with the highest volume in the 2 weeks before and after the quit date. For smokers who were not ready to quit in the next 30 days, 31 novel messages were developed by an expert team of primary care physicians (PCPs), a tobacco cessation counselor, a mobile health manager, and a behavioral scientist.

**Table 1:** Sample messages from GetReady2Quit (R2Q).

Type		Example	Reference
Opt in		Reply “yes” to participate in the R2Q Text Connect program offered to you by your doctor.	—
**Campaign for smokers ready to quit in the next 30 days**			
	Behavioral advice	Next time you have the urge to smoke, try and resist for 5 minutes. Or skip the cigarette entirely. Think of it as practice for quit day!	[33]
	Motivational messages	Need motivation? Make a list of your reasons for quitting. Put it someplace you can see every day. Keep thinking about why you want to quit.	[33]
	Educational messages	Lung capacity increases by 30% after a few weeks without cigarettes! Ride your bike or take a walk. Put your healthy lungs to good use!	[33]
	Smoking status	Are you still smokefree? Reply: YES or NO.	[33]
	Keywords	Text your supporters and remind them of the big day. Make sure they are there for you. Text back CRAVE, MOOD, or SLIP for more support anytime.	[33]
**Campaign for smokers not ready to quit in the next 30 days**			
	Motivational messages	Write down your reasons to quit. Put your reasons someplace where you will see them when you smoke, like in your car, your kitchen, or at your computer.	[35]
	Practice quit attempt	A practice quit attempt is a few hours or days when you don’t smoke to learn how you will feel when quit for real. Try it this week!	[36]
	Readiness to quit	Are you ready to quit for good? If yes, reply with the date you would like to quit on, in this format: MMDD, for ex: 0513 for May 13. If not, reply WAIT.	—

Content included 16 motivational messages to encourage individuals to identify personal reasons for change and internal motivations to quit [35,37]. Fifteen messages encouraged smokers to try a practice quit attempt explained as an attempt to not smoke for hours or days without a commitment to stop for good [36]. Practice quit attempts can increase motivation and self-efficacy [36,38]. Smokers not ready to quit were sent three to five messages per week. At the end of this message campaign, users were asked again if they were ready to quit in the next 30 days. Those that were ready were sent the QuitNowTXT messages. Those that were not ready were sent a final recommendation to contact their doctor or the state quitline.

### Procedures

Between March 2014 and June 2015, PCPs were asked to screen potentially eligible patients. The PCP-approved patients were sent an opt-out letter (Multimedia Appendix 1), informing them about the purpose of the feasibility study, content of the R2Q text messaging program, and that they would be sent a text message in the next week unless they called to opt out. Patients who did not opt out were sent a single text message inviting them to opt in to the R2Q program (Table 1). Opting in implied consent. Participants were sent four text message queries assessing nicotine dependence, readiness to quit, and quit date. Ethical approval was obtained from the Partners Healthcare, Inc Institutional Review Board.

### Statistical Analysis

We compared R2Q enrollees, those who opted in following the proactive text message, with patients who were eligible and invited but who did not enroll in terms of demographics, primary insurance, and comorbidities. We used portions of the Reach Effectiveness Adoption Implementation Maintenance (RE-AIM) methodology [31] to measure program reach, engagement, and adoption. Reach was the proportion of users sent a proactive text invitation who opted in. Engagement was measured as sending one or more keywords to the server. Adoption was defined as days in the text messaging program before the participant texted “STOP” or failed to respond to an assessment message. We compared engagement and adoption by readiness to quit using unadjusted *t* tests, Wilcoxon rank sum tests, and chi-square tests.

## Results

**Figure 1:**
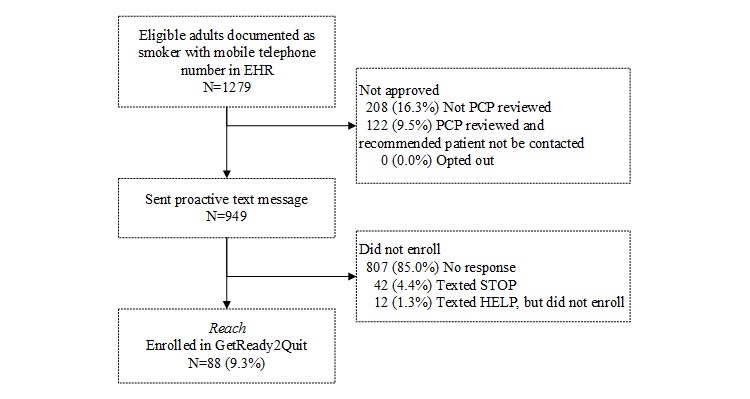
GetReady2Quit patient enrollment flow. Eligible patients were adults (>18 years) listed as current smoker in their electronic health record (EHR) with English listed as primary language. PCP: primary care physician.

We identified 1279 adults who met our inclusion criteria. Of these, 949 patients were reviewed and approved by their PCP for recruitment and 88 patients enrolled by opting in to the program after a single recruitment text for a reach of 9.3% (Figure 1).

Enrollees were more likely to be female and were less likely to have cardiovascular disease, but did not otherwise differ from eligible patients who were sent the opt-in text but who did not enroll (Table 2). Of the 88 enrollees, 67 (76%) completed all query messages about readiness to quit and nicotine dependence. Seven enrollees (11%) had already quit, 19 (28%) were not ready to quit, and 41 (61%) were ready to quit in the next 30 days. Of the 60 current smokers, median time in the program was 9 days (interquartile range [IQR] 2-32 days). Fifteen of 60 (25%) current smokers engaged with the program by texting keyword messages (eg, CRAVE, MOOD, or SLIP). Program time and engagement did not differ by readiness to quit (Table 3). However, compared to smokers not ready to quit, those ready to quit received more messages (median 18, IQR 14-40 vs median 12, IQR 7-44, *P*=.04).

**Table 2:** Characteristics of eligible participants by GetReady2Quit enrollment status.

Characteristics		Enrolled (N=88)	Did not enroll (N=861)	χ^2^ (df)	*t*_944_	*P*
**Demographics**						
	Gender (female), n (%)	54 (61.4)	413 (48.0)	5.7 (1)	–	.02
	Race (white), n (%)	78 (89.7)	754 (87.8)	0.3 (1)	–	.61
	Age (years), mean (SD)	47 (12)	48 (14)	–	0.7	.50
**Primary insurance, n (%)**		–	–	1.8 (3)	–	.61
	Commercial insurance	52 (59.8)	458 (53.3)	–	–	–
	Medicaid	18 (20.7)	233 (27.1)	–	–	–
	Medicare	15 (17.2)	149 (17.4)	–	–	–
	Self-pay	2 (2.3)	19 (2.2)	–	–	–
**Comorbidities, n (%)**						
	Cardiovascular disease	3 (3.4)	87 (10.1)	4.2 (1)	–	.04
	Diabetes mellitus	11 (12.5)	88 (10.2)	0.4 (1)	–	.51
	Hypertension	22 (25.0)	255 (29.6)	0.8 (1)	–	.36
	Chronic kidney disease	1 (1.1)	13 (1.5)	0.1 (1)	–	.78
	Depression	2 (2.3)	25 (2.9)	0.1 (1)	–	.74

**Table 3:** Characteristics of enrolled smokers by readiness to quit.

Characteristics	Ready to quit (n=41)	Not ready to quit (n=19)	χ^2^_1_	*t*_48_	*z*	*P*
Cigarettes per day, mean (SD)	15 (7)	15 (5)	–	–0.1	–	.92
Time to first cigarette (<30 minutes), n (%)	32 (80)	10 (77)	0.1	–	–	.81
Messages received, median (IQR^a^)	18 (14-40)	12 (7-44)	–	–	–2.0	.04
Engagement (program days), median (IQR)	16 (3-31)	4 (1-35)	–	–	–0.8	.40
Adoption (texted a keyword), n (%)	13 (32)	2 (11)	3.1	–	–	.08

^a^IQR: interquartile range.

## Discussion

### Comparison With Prior Work

This study evaluated a proactive tobacco cessation intervention that reached out to patients by text message. It shows promise as a low-cost, scalable intervention for primary care populations. Program reach at 9.3% was comparable to other proactive care models for smokers that used more intensive outreach methods, including up to 15 outreach telephone calls [24]. Similar to telephone outreach programs, both smokers ready to quit and those not ready to quit enrolled [27]. These results support the feasibility of future work to design and test a proactive text messaging intervention targeting primary care patients.

Text messaging programs originating from the physicians’ office may leverage the influence physicians have on smokers [18]. Individuals most often look to their own health care systems for online health information [39]; therefore, trust in their health care providers may make health-promoting advice more potent if it is coming from their physicians’ office. This trust may also encourage even unmotivated smokers to engage in health-promoting activities sent to them by their physicians’ office.

Our single message enrollment process was simpler than recruitment used by other proactive care models [20-26]. The low intensity may have been appealing to both smokers not ready to quit and those busy managing other chronic diseases, who may not have time or interest in more complex interventions. Indeed, except for cardiovascular disease, patients with comorbid chronic diseases were no less likely to opt in. Integrating the program with other optional cessation services, such as pharmacotherapy, may increase the program’s appeal and improve reach and effectiveness. Future work will need to explore ways for text messaging to be integrated with other cessation services available to primary care patients who smoke.

### Limitations

In this pilot study, we did not have enough resources to assess smoking outcomes or receipt of text messages. Therefore, we could not account for invalid telephone numbers or failed message delivery. If these are considered, the uptake of the program following receipt of the proactive text may have been even higher.

### Conclusions

A proactively delivered text messaging program targeting primary care patients who smoke reached as many smokers with a single text as more intense and costly telephone call- or mailed-based proactive outreach methods. This method engaged both smokers ready to quit and those not ready to quit and shows promise as part of a proactive care model for addressing smoking in primary care populations.

## Appendix

Multimedia Appendix 1 Opt-out patient letter.

## Abbreviations

EHR: electronic health record
IQR: interquartile range
PCP: primary care physicians
R2Q: GetReady2Quit
