# Supplementary material for: Feasibility of a Proactive Text Messaging Intervention for Smokers in Community Health Centers: Pilot Study
Source: JMIR Form Res. 2018 May 31;2(1):e11. doi: 10.2196/formative.9608 (PMC6261471; doi:10.2196/formative.9608)
Supplement: Multimedia Appendix 1 [file formative_v2i1e11_app1.pdf]

R2Q: GetReady2Quit

«AddressBlock» <<DATE>>

«GreetingLine»

I would like to offer you the chance to join a research study of the GetReady2Quit text messaging program (R2Q). I am sending this letter because our records indicate that you may be a smoker. The R2Q program sends text messages to your phone to help you try to quit smoking or with helpful tips if you are not yet ready to quit. R2Q is offered by the Partners Center for Connected Health, the Tobacco Research and Treatment Center and Partners HealthCare System, Inc. ("Partners"). I encourage you to join the study.

R2Q is designed to support, not replace, your relationship with me. If you choose not to join, it will not affect your care with me. Do not use R2Q for any medical issues. Call 911 or call us here at the clinic in the event of a medical emergency.

Next week, you will receive a text message inviting you to enroll in R2Q. Your participation in R2Q is voluntary.

To enroll, you can either:

(1) Reply 'YES' by text to that message OR
(2) Text the word 'QUIT' to XXXXX to enroll from any mobile phone at any time

If you decide later that you no longer want to receive the R2Q messages, you can end the messages at any time by texting the word 'STOP' to XXXXX.

If you do not want to enroll at all or if you are not a smoker,

(1) Simply do not reply to that message, we will only send it once OR
(2) Reply 'NO' to that message OR
(3) Call XXX-XXX-XXXX, to let us know that you don't want to participate

If you have questions about R2Q, contact the study staff at XXX-XXX-XXXX.

Sincerely,

<<PCP field>>

R2Q Terms and conditions

By enrolling:

- You agree to receive between 25 and 60 messages each month for up to 3 months. If you have unlimited texting, these messages will be free, the messages are paid for by Partners. <u>If you pay to receive each text message, your cell phone carrier will charge you to receive the R2Q messages, up to a maximum of $12 each month.</u>
- You agree to receive text messages about smoking. Your responses will be confidential.
- You agree to have your data reviewed for quality improvement. The Partners Center for Connected Health, the text messaging vendor, and I will also have access to the R2Q messages.
- You agree to be contacted by research staff by phone during the study for customer service, quality improvement, or to invite you to participate in focus groups about the R2Q. You are not required to respond to any of these requests for feedback, everything is voluntary.
- Federal law requires that Partners protect the privacy of health information that identifies you. Partners uses encryption technology to protect your privacy. Program staff will also not include any health information in any voice mail message to you.
